# Association between low hemoglobin, clinical measures, and patient-reported outcomes in patients with rheumatoid arthritis: results from post hoc analyses of three phase III trials of sarilumab

**DOI:** 10.1186/s13075-022-02891-x

**Published:** 2022-08-25

**Authors:** Andrea Rubbert-Roth, Daniel E. Furst, Stefano Fiore, Amy Praestgaard, Vivian Bykerk, Clifton O. Bingham, Christina Charles-Schoeman, Gerd Burmester

**Affiliations:** 1 Division of Rheumatology, Cantonal Hospital, St. Gallen, Switzerland; 2 University of California, Los Angeles, CA USA; 3 Sanofi, Bridgewater, NJ USA; 4 Hospital for Special Surgery, New York, NY USA; 5 Johns Hopkins University, Baltimore, MD USA; 6 Department of Rheumatology and Clinical Immunology, Charité – Universitätsmedizin Berlin, Freie Universität Berlin and Humboldt-Universität Berlin, Berlin, Germany

**Keywords:** Rheumatoid arthritis, Sarilumab, Patient-reported outcomes, Anemia, Hemoglobin, Radiographic outcomes, TARGET, MOBILITY, MONARCH

## Abstract

**Background:**

Anemia is common in patients with rheumatoid arthritis (RA). Higher hemoglobin (Hb) levels may be associated with better clinical outcomes and patient-reported outcomes (PROs). To assess this hypothesis, we conducted two post hoc analyses in three sarilumab phase III studies: TARGET, MOBILITY, and MONARCH.

**Methods:**

Pooled data from combination therapy from placebo-controlled MOBILITY (sarilumab + methotrexate) and TARGET (sarilumab + conventional synthetic disease-modifying antirheumatic drugs [csDMARDs]) and monotherapy data from active-controlled MONARCH (sarilumab vs. adalimumab) studies were included. Associations between Hb levels and clinical measures and PROs were assessed over 24 weeks. The mean changes from baseline in clinical outcomes and PROs (to week 24) and radiographic outcomes (to week 52) were evaluated between low and normal Hb levels (based on the World Health Organization [WHO] criteria).

**Results:**

From TARGET, MOBILITY, and MONARCH, 546, 1197, and 369 patients, respectively, were stratified according to Hb levels (low vs. normal). Over 24 weeks, higher Hb levels were found to be consistently associated with better clinical outcomes and PROs in combination therapy and monotherapy groups and were more pronounced among the patients treated with sarilumab than those treated with placebo and adalimumab. The mean change from baseline to week 24 in clinical efficacy measures and PROs was similar in patients with low vs. normal Hb at baseline. Differences between sarilumab and/or adalimumab, for all outcomes, were larger for low Hb subgroups. In MOBILITY, by week 52, the inhibition of progression of structural damage (assessed via Modified Total Sharp Score [mTSS]) was 84% (sarilumab 200 mg) and 68% (sarilumab 150 mg) vs. placebo in patients with low Hb and 97% (sarilumab 200 mg) and 68% (sarilumab 150 mg) vs. placebo in patients with normal Hb. Similar results were observed for other radiographic outcomes.

**Conclusions:**

In these post hoc analyses, a consistent relationship was observed between higher Hb levels and better clinical outcomes and PROs in patients with RA. Irrespective of the baseline Hb levels, sarilumab treatment was associated with improvements in clinical measures and PROs over 24 weeks (improvements were more pronounced than those with adalimumab treatment) and mitigation of joint damage progression over 52 weeks.

**Trial registration:**

ClinTrials.gov NCT01061736, NCT01709578, and NCT02332590

**Supplementary Information:**

The online version contains supplementary material available at 10.1186/s13075-022-02891-x.

## Introduction

Rheumatoid arthritis (RA) is a chronic, systemic autoimmune disease that, when untreated, progressively results in destruction and disability of the joints in 65 to 70% of patients and is associated with significant effects on social health and economic challenges. If left untreated, RA results in shortened life expectancy [1–4]. The global prevalence of RA in the adult population is approximately 0.3 to 1.0% [5].

Chronic inflammatory conditions may exhibit various hematologic abnormalities such as anemia, which develops in 15 to 47% [6–8] of patients with RA. The etiology of anemia in RA is complex and multifactorial [9]. Proinflammatory cytokines, including tumor necrosis factor (TNF)-alpha, interleukin (IL)-1, IL-6, and IL-10, play important roles in RA-associated anemia [10]. IL-6 may exert its effect by inducing hepcidin, which is a key regulator of body iron homeostasis [11]. The circulating serum hepcidin level is reported to be increased in RA patients with anemia of chronic disease [12, 13]. In such patients, anemia is associated with high disease activity [14], erosive arthritis [15], and an increased risk of mortality [7]. Hemoglobin (Hb) levels may also correlate with radiographic outcomes [7, 16].

The use of an anti-IL-6 receptor (IL-6R) monoclonal antibody in RA patients with anemia has been reported previously and has provided evidence for greater improvement in Hb levels compared to the control group [17, 18].

Two monoclonal antibodies that target IL-6 signaling, sarilumab and tocilizumab (TCZ), are approved for the treatment of RA. TCZ has been shown to improve anemia in patients with RA [17–19].

Sarilumab, an IL6-R antagonist, is approved for the treatment of adult patients with moderate-to-severe RA with an inadequate response or intolerance to one or more disease-modifying antirheumatic drugs (DMARDs) [20–22].

We hypothesized that, in RA patients, higher Hb levels are associated with better outcomes and that this association would be more pronounced among the patients treated with sarilumab. To evaluate this hypothesis, we conducted two post hoc analyses to assess the relationship between Hb and clinical outcomes and Hb and radiographic progression in three phase III studies of sarilumab, TARGET (NCT01709578), MOBILITY (NCT01061736), and MONARCH (NCT02332590).

## Methods

### Study design

Details of the phase III study designs have been described previously [21, 23, 24]. In brief, the MOBILITY study was conducted to investigate the efficacy and safety of sarilumab (vs. placebo) in combination with methotrexate (MTX) in patients with moderate-to-severe active RA and an inadequate response to methotrexate (MTX-IR) through 52 weeks. The TARGET study investigated the efficacy and safety of sarilumab (or placebo) in combination with background conventional synthetic disease-modifying antirheumatic drugs (csDMARDs) in patients with moderate-to-severe RA who were intolerant of, or who had an inadequate response to, tumor necrosis factor (TNF) inhibitors (TNFi-INT/IR) through 24 weeks. The MONARCH study compared the 24-week efficacy and safety of sarilumab monotherapy with adalimumab monotherapy in biologic DMARD-naïve patients with moderate-to-severe active RA who were intolerant of or had an inadequate response to MTX (MTX-INT/IR).

For the post hoc analysis that evaluated the relationship between Hb levels and clinical efficacy measures and PROs, the data sets included pooled data from combination therapy from TARGET and MOBILITY and monotherapy data from MONARCH through 24 weeks. The other post hoc analysis evaluated the proportion of RA patients with low Hb at baseline and whether such patients were at an increased risk of joint damage progression compared to those with normal baseline Hb levels. The baseline characteristics and radiographic outcomes in MOBILITY were analyzed by baseline Hb category (low vs. normal) [25].

The three protocols were approved by the appropriate ethics committees/institutional review boards, and each patient provided written informed consent before participating in the study. The studies were conducted in compliance with institutional review board regulations, the International Conference on Harmonization Guidelines for Good Clinical Practice, and the Declaration of Helsinki.

### Assessments and endpoints

In all the three studies, Hb levels were defined as low (85–120 g/L [female patient]/85–130 g/L [male patient]) vs. normal (120–155 g/L [female patient]/130–175 g/L [male patient]). Hb levels were assessed at baseline to week 24 in all three studies and at baseline to week 52 in MOBILITY.

Endpoints were the mean change from baseline through 24 weeks in clinical assessments including clinical disease activity index (CDAI), C-reactive protein (CRP), Disease Activity Score 28-joint count-CRP (DAS28-CRP), physician-assessed 28 swollen and tender joint counts (SJC28 and TJC28), Physician Global Assessment (MDGA) and patient-reported assessments including the patient global assessment (PtGA), pain, and morning stiffness using visual analog scale (pain-VAS and morning stiffness-VAS using 0–100 mm VAS), and Functional Assessment of Chronic Illness Therapy-Fatigue (FACIT-F).

Radiographic outcomes assessed in this study were Modified Total Sharp Score (mTSS), joint space narrowing (JSN, sum of JSN scores from 42 joints), and erosion score (sum of erosion scores from 44 joints). Endpoints were the changes in mTSS, JSN, and erosion score from baseline to 24 weeks and 52 weeks.

### Statistical methods

**Fig. 1:**
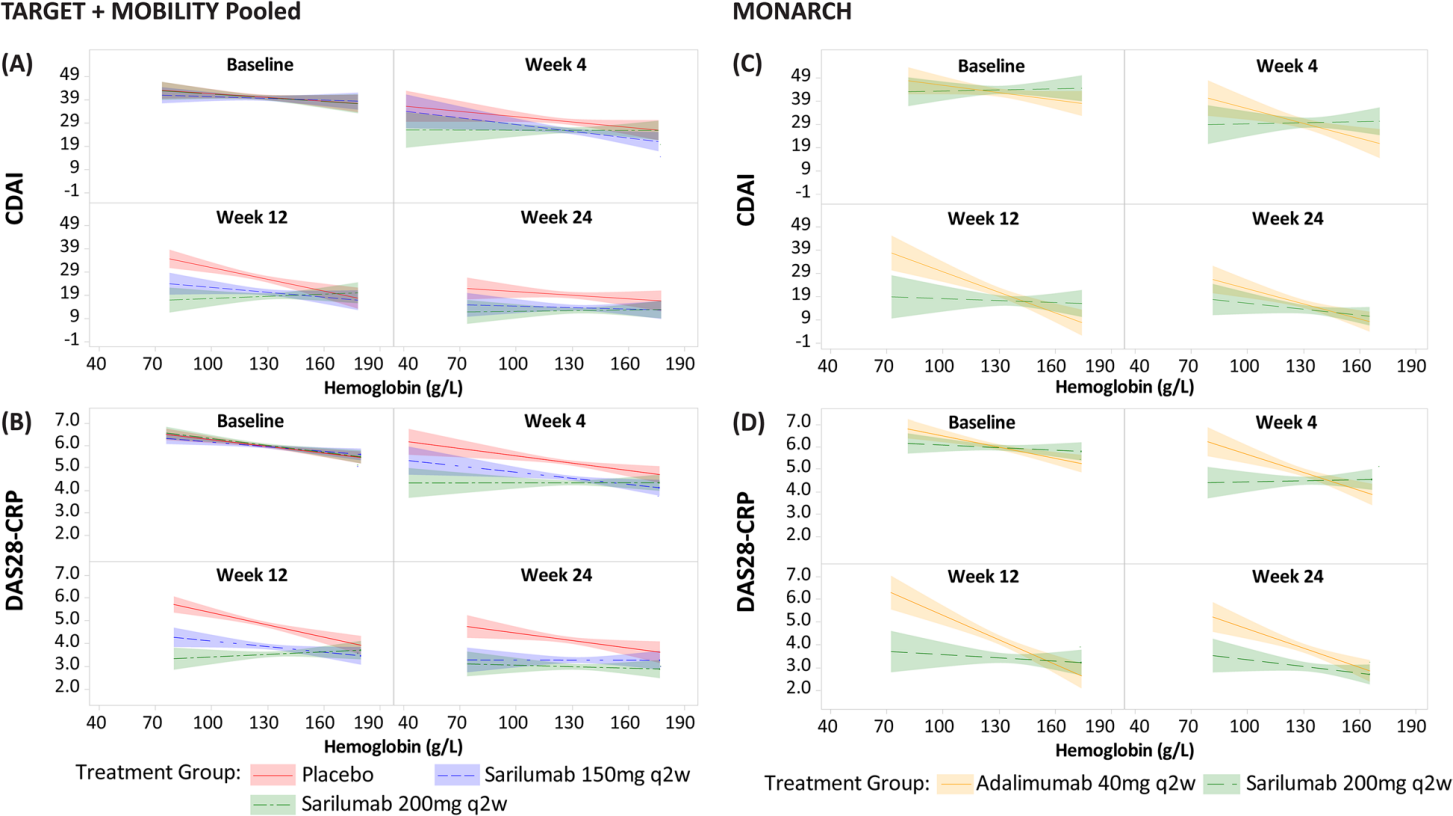
Relationship between Hb levels and clinical efficacy outcomes: CDAI and DAS-28 CRP over time. Slicefit plots of CDAI and DAS-28 CRP (*Y*-axis) against hemoglobin (g/L) (*X*-axis) by a visit from baseline to week 24. Patients with non-missing CRP (mg/L) and hemoglobin (g/L) values were considered. Each panel in the regression model shows the relationship between an outcome and Hb level at a given point in time, stratified by treatment. The lines, with shading, depict the treatment-specific regression coefficient (i.e., slope) and 95% CI associated with the linear regression in which Hb is the independent variable and the outcome is the dependent variable. CDAI, Clinical Disease Activity Index; CRP, C-reactive protein; DAS, Disease Activity Scores; Hb, hemoglobin; q2w, every 2 weeks

**Fig. 2:**
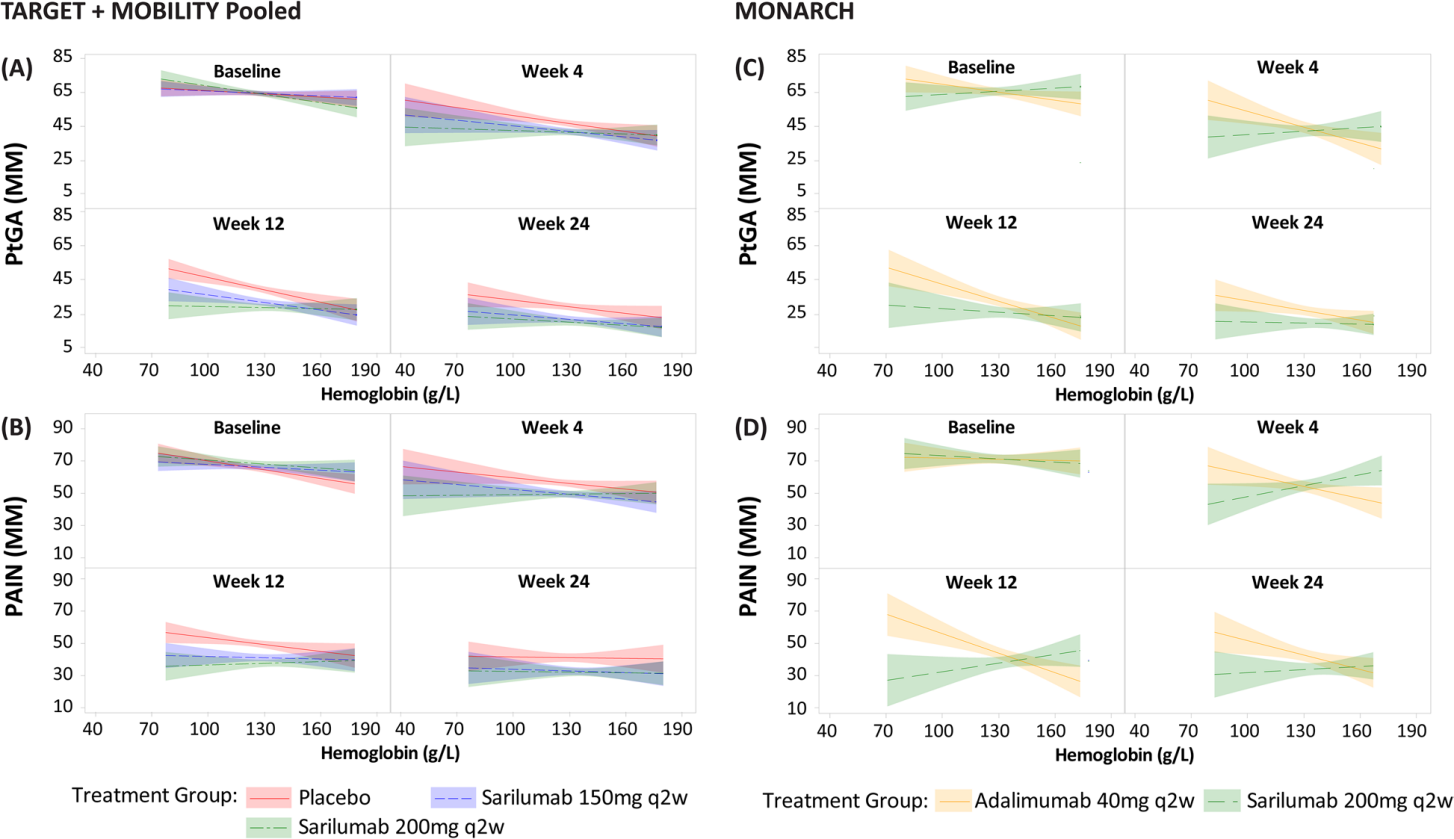
Relationship between Hb levels and clinical efficacy outcomes: PtGA and pain VAS over time. Slicefit plots of PtGA (MM) and pain VAS (MM) (*Y*-axis) against hemoglobin (g/L) (*X*-axis) by a visit from baseline to week 24. Each panel in the regression model shows the relationship between an outcome and Hb level at a given point in time, stratified by treatment. The lines, with shading, depict the treatment-specific regression coefficient (i.e., slope) and 95% CI associated with the linear regression in which Hb is the independent variable and the outcome is the dependent variable. Hb, hemoglobin; pain VAS, pain visual analog scale; PtGA, Patient Global Assessment; q2w, every 2 weeks

All analyses that were based on low-Hb and normal-Hb level patient groups were conducted post hoc. Bivariate linear regression analysis evaluated the relationships between Hb levels and outcomes at baseline and weeks 4, 8, 12, 20, and 24. In these regression models, the dependent variable was a clinical efficacy or PRO endpoint, and independent variables were treatment, Hb group, and interaction between treatment and Hb group (as indicated in Figs. 1 and 2). Categorical variables were summarized using the number and percentage (%) of patients. The mean changes from baseline in clinical efficacy measures and PROs were summarized as the mean (standard deviation [SD]) values. Forest plots were used to depict the mean difference between baseline and week 24 and week 52 for patients with low and normal baseline Hb by treatment arm and for each radiographic endpoint. Rank analysis of covariance (ANCOVA) models including prior biologic use, region, and baseline radiographic values as covariates were applied to assess the impact of sarilumab on the radiographic outcomes. A nominal *P* value of < 0.05 was considered significant in this analysis.

## Results

### Patients’ baseline characteristics and disposition

Across the three phase III studies, 186 patients in TARGET (*N* = 546), 414 patients in MOBILITY (*N* = 1197), and 92 patients in MONARCH (*N* = 369) had low Hb levels (Table 1). In all the evaluated study groups, low Hb was more common in female patients vs. male patients (TARGET [86.0% vs. 14.0%], MOBILITY [85.8% vs. 14.3%], and MONARCH [84.8% vs. 15.2%]). In MOBILITY, low Hb was more common in Asians (14% vs. 5%), young patients (mean age 49 vs. 51 years), and those with lower body weight (mean 69 vs. 77 kg); all *P* values < 0.01. In MOBILITY, there was a significant difference in CRP between low baseline Hb and normal baseline Hb groups (mean: 30.2 [SD: 28.5] vs. 17.3 [18.5] mg/L; *P* < 0.0001). Differences in CRP between low baseline Hb and normal baseline Hb groups were also observed in TARGET and MONARCH studies.

**Table 1:** Patients’ demographics and baseline disease characteristics

	TARGET (TNFi-INT/IR)		MOBILITY (MTX-IR)		MONARCH (MTX-INT/IR)	
	Low Hb (***N*** = 186)	Normal Hb (***N*** = 360)	Low Hb (***N*** = 414)	Normal Hb (***N*** = 783)	Low Hb (***N*** = 92)	Normal Hb (***N*** = 277)
Age, years	52.1 (13.9)	53.4 (11.5)	49.2 (12.6)	51.3 (11.0)	50.0 (13.3)	53.0 (11.9)
Female, *n* (%)	160 (86.0)	287 (79.7)	355 (85.8)	622 (79.4)	78.0 (84.8)	229 (82.7)
Male, *n* (%)	26 (14.0)	73 (20.3)	59 (14.3)	161 (20.6)	14 (15.2)	48 (17.3)
Weight, kg	74.7 (20.9)	80.0 (21.6)	69.5 (16.9)	76.9 (18.8)	69.3 (19.8)	73.0 (16.1)
BMI, kg/m^2^	28.7 (7.5)	30.0 (7.0)	26.7 (6.0)	29.1 (6.4)	26.2 (7.1)	27.5 (5.7)
Race, *n* (%)						
White	118 (63.4)	270 (75.0)	336 (81.2)	695 (88.8)	82 (89.1)	253 (91.3)
Black	14 (7.5)	6 (1.7)	11 (2.7)	17 (2.2)	1 (1.1)	3 (1.1)
Asian/Oriental	3 (1.6)	2 (0.6)	58 (14.0)	40 (5.1)	5 (5.4)	6 (2.2)
Others	51 (27.4)	82 (22.8)	9 (2.2)	31 (4.0)	4 (4.4)	15 (5.4)
Duration of RA since diagnosis, years	12.2 (9.6)	12.0 (9.3)	8.6 (8.0)	9.3 (7.8)	6.3 (7.3)	7.7 (8.2)
Anti-CCP [*n* (%)] positive	147 (79.9)	275 (77.3)	350 (84.8)	686 (88.0)	73 (81.1)	199 (74.3)
Rheumatoid factor [*n* (%)] positive	147 (79.5)	262 (73.4)	344 (83.3)	665 (85.5)	63 (70.0)	172 (64.4)
TJC28 (0–28)	17.1 (7.4)	15.8 (6.6)	16.0 (6.7)	15.6 (6.6)	16.7 (6.4)	16.7 (6.2)
SJC28 (0–28)	14.5 (6.3)	13.1 (5.9)	12.2 (5.7)	11.8 (5.4)	13.3 (5.8)	12.8 (5.5)
HAQ-DI (0–3)	1.9 (0.6)	1.7 (0.6)	1.7 (0.7)	1.6 (0.6)	1.7 (0.5)	1.6 (0.6)
CRP, mg/L*	36.9 (30.5)	21.6 (21.4)	30.2 (28.5)	17.3 (18.5)	35.0 (35.0)	16.0 (21.5)
CDAI	46.0 (14.0)	42.4 (12.4)	41.2 (12.4)	40.1 (12.2)	43.6 (12.9)	42.8 (11.8)
DAS28-CRP	6.5 (1.0)	6.1 (0.9)	6.1 (0.9)	5.9 (0.9)	6.3 (0.9)	5.9 (0.9)
Pain-VAS, mm	75.9 (18.2)	70.7 (18.8)	66.2 (20.2)	64.7 (21.3)	71.5 (17.9)	71.5 (19.1)
Morning stiffness VAS, 0–100 mm	72.4 (22.0)	67.7 (23.1)	Not Available	Not Available	68.6 (20.5)	69.7 (20.2)
FACIT-Fatigue	22.8 (10.0)	23.8 (10.8)	25.3 (10.4)	27.0 (10.1)	23.0 (9.2)	24.0 (9.8)

All values are mean (SD), unless specified otherwise

*N* number of patients in each study group with low or normal Hb subgroup, *BMI* body mass index, *CCP* cyclic citrullinated peptide, *CRP* C-reactive protein, *CDAI* Clinical Disease Activity Index, *DAS* Disease Activity Scores, *DAS28-CRP* Disease Activity Score 28-joint count-C-reactive protein, *FACIT-F* Functional Assessment of Chronic Illness Therapy-Fatigue, *HAQ-DI* Stanford Health Assessment Questionnaire-Disability Index, *Hb* hemoglobin, *MTX-INT/IR* intolerant of, or an inadequate response to, MTX, *MTX-IR* An inadequate response to methotrexate, *RA* rheumatoid arthritis, *SD* standard deviation, *SE* standard error, *SJC28* Swollen 28-Joint Count, *TJC28* Tender 28-Joint Count, *TNFi-INT/IR* intolerant of, or an inadequate response to, tumor necrosis factor inhibitors, *VAS* visual analog scale

*Significant difference in CRP between low and normal baseline Hb groups in MOBILITY study (*P* < 0.0001)

Duration of RA and other factors were similar between patients with low and normal baseline Hb in all the evaluated study groups (TARGET, MOBILITY, and MONARCH). Prior RA medication use was also similar between the patients with low and normal baseline Hb in all study groups (Suppl Table 1).

### TARGET MOBILITY Pooled and MONARCH: mean change in Hb levels over time

During the TEAE period (from baseline to 52 weeks), the mean changes in Hb (g/L) were as follows: placebo—mean change: − 0.6 (95% CI: − 2.15–0.87); sarilumab 150 mg—mean change: 9.1 (95% CI: 7.76–10.42); and sarilumab 200 mg—mean change: 10.0 (95% CI: 8.72–11.29) (Table 2).

**Table 2:** Mean change in Hb levels over time by treatment arm (TARGET, MOBILITY, and MONARCH)

Hb (g/L)	Baseline	Mean change from baseline to 24 weeks	Mean change from baseline to 52 weeks
**TARGET**			
Placebo + DMARD	126.6	− 0.3	–
Sarilumab 150 mg q2w + DMARD	128.1	+ 8.0	–
Sarilumab 200 mg q2w + DMARD	125.7	+ 9.0	–
**MOBILITY**			
Placebo + MTX	127.0	− 0.3	− 0.6
Sarilumab 150 mg + MTX	125.6	+ 7.9	+ 9.1
Sarilumab 200 mg + MTX	126.6	+ 8.2	+ 10.0
**MONARCH**			
Adalimumab 40 mg q2w	129.5	+ 1.1	–
Sarilumab 200 mg q2w	130.1	+ 6.0	–

Mean changes in Hb levels were reported at baseline, week 24, and week 52 (MOBILITY-B only)

*DMARD* disease-modifying antirheumatic drugs, *Hb* hemoglobin, *q2w* every 2 weeks, *SD* standard deviation

### TARGET+MOBILITY Pooled and MONARCH: relationship between Hb and multiple endpoints from baseline through week 24

From baseline to week 24, higher Hb levels were consistently associated with better outcomes, irrespective of the study groups, in TARGET+MOBILITY Pooled and MONARCH; however, we observed that this association was more pronounced among the patients treated with sarilumab than those treated with placebo and/or adalimumab. Moreover, a steady disconnect between Hb and clinical outcomes was observed in sarilumab-treated patients through week 24 and not observed in placebo or adalimumab (Fig. 1).

Disassociation between Hb level and endpoints was consistent between the two evaluated study groups (TARGET+MOBILITY Pooled) and MONARCH through week 24 across multiple additional clinical endpoints including PtGA and pain-VAS (Fig. 2), TJC28 and SJC28 (Suppl. Fig. 1), and FACIT-F and morning stiffness-VAS (Suppl. Fig. 2).

Scatterplots from MONARCH were generated to evaluate the improvement in CDAI by Hb status (low-left of the WHO cutoff lines; normal-right of WHO cutoff lines) in patients treated with sarilumab (panel A) or adalimumab (panel B) over 24 weeks (Suppl. Fig. 3). Improvement in CDAI by Hb status was higher with sarilumab (68%; panel A) vs. adalimumab (61%; panel B).

By week 24, the proportion of patients with improvement in CDAI and Hb (“responders” defined as patients with a CDAI improvement from baseline of ≥ 50% and Hb as ≥ 120 g/L for women or Hb ≥ 130 g/L for men) was 71.2% (*n* = 111/156) in the sarilumab group and 60.8% (*n* = 90/148) in the adalimumab group (Suppl. Table 2).

For TARGET+MOBILITY Pooled data, the improvement in CDAI and Hb was more pronounced in patients receiving sarilumab 150 mg and 200 mg q2w vs. placebo (Suppl. Fig. 4). By week 24, the proportion of responders was 42.9% (*n* = 145/338) in the placebo group, 64.6% (*n* = 227/429) in the sarilumab 150 mg group, and 71.6% (*n* = 313/437) in the sarilumab 200 mg group (Suppl. Table 3).

### TARGET+MOBILITY Pooled and MONARCH: mean change in PROs from baseline to week 24

**Fig. 3:**
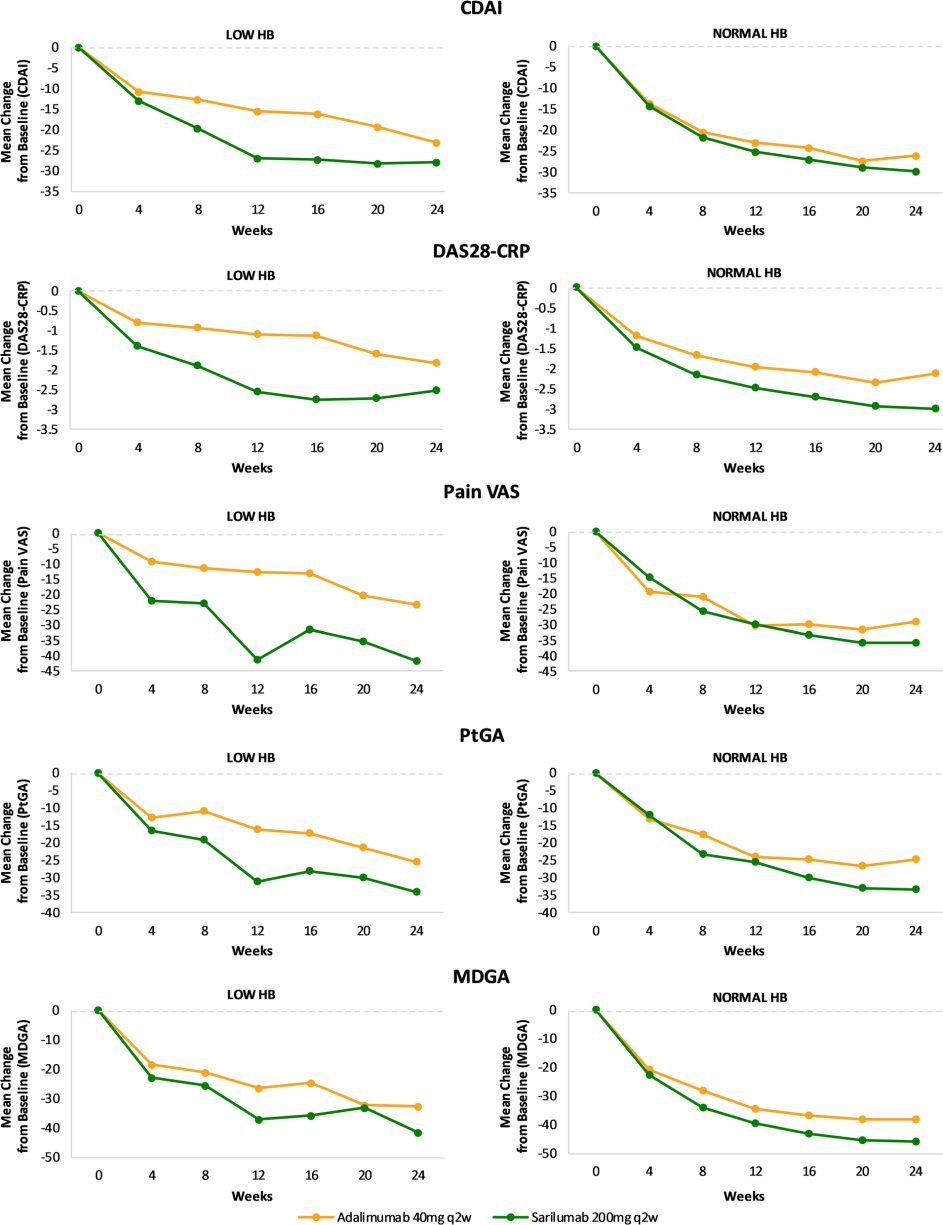
Mean change in treatment outcomes over time (stratified by Hb level at baseline)—MONARCH. CDAI, Clinical Disease Activity Index; CRP, C-reactive protein; DAS28-CRP, Disease Activity Score-28 for Rheumatoid Arthritis with CRP; Hb, hemoglobin; PtGA, Patient Global Assessment; MDGA, Physician Global Assessment; q2w, every 2 weeks; VAS, visual analog scale

The mean change in CDAI, DAS-28-CRP, and MDGA at week 24 was similar in both low Hb and normal Hb subgroups. The results for the PROs (pain-VAS, PtGA) in MONARCH (Fig. 3) and TARGET+MOBILITY are shown in Suppl. Fig. 5. The effect size (difference between sarilumab and comparator adalimumab) was larger for the low Hb subgroup in MONARCH, but there were no obvious conclusions regarding the relationship between Hb and PROs in TARGET +MOBILITY (Suppl Fig. 5). Similarly, the effect size observed for TJC28, SJC28, FACIT-F, and morning stiffness-VAS in the low Hb subgroup were modest with the combination therapy group (Suppl. Fig. 6) compared to the monotherapy group (Suppl. Fig. 7).

### MOBILITY: mean change in radiographic outcomes (from baseline to week 52)

#### Modified Total Sharp Score

**Fig. 4:**
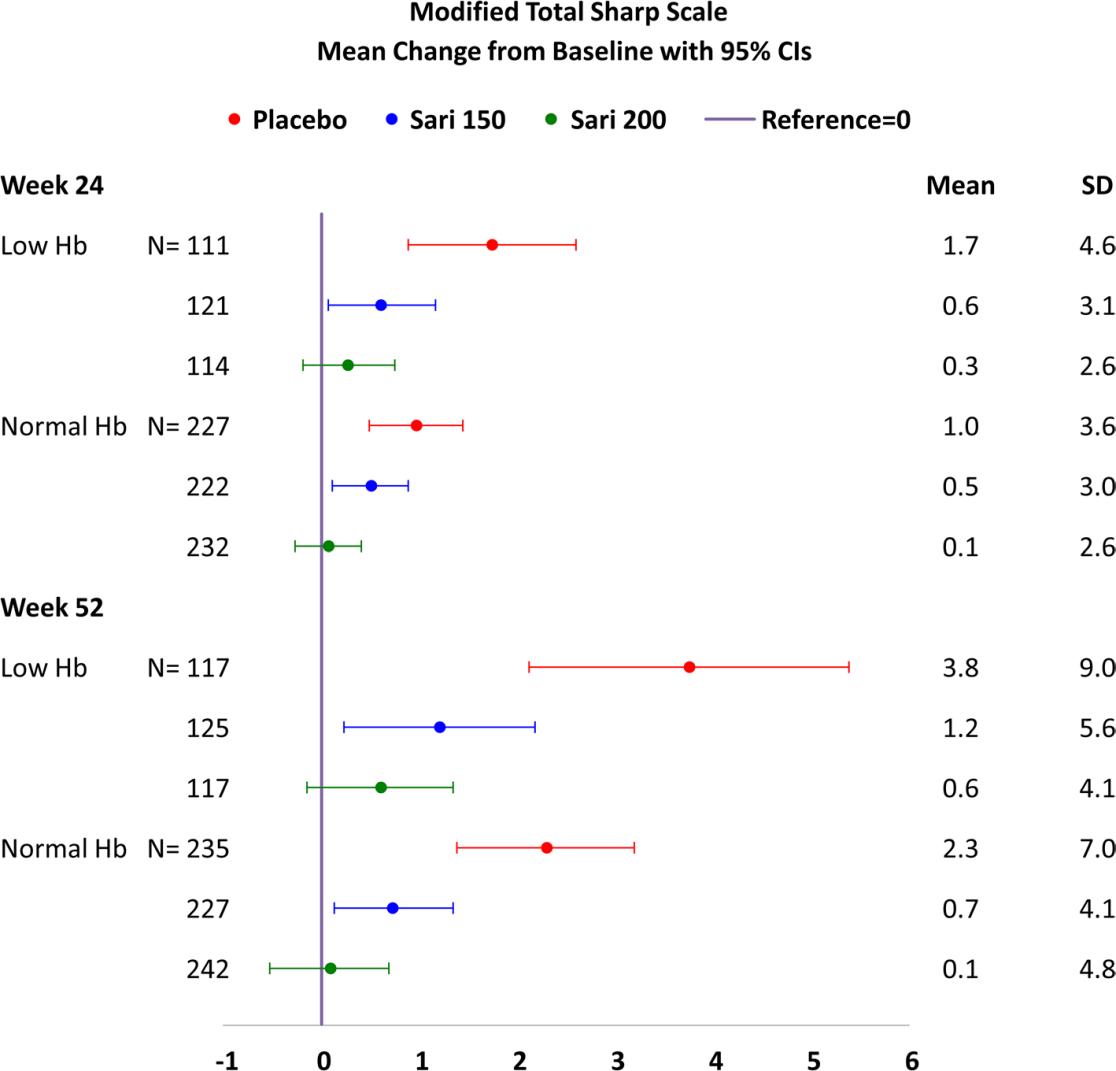
Mean change in mTSS from baseline at week 24 and week 52 (95% CI)—MOBILITY. All patients received weekly MTX, and sarilumab or placebo were administered q2w. At week 52, low Hb: *P* < 0.001 by rank ANCOVA for each sarilumab dose vs. placebo; normal Hb: rank ANCOVA *P* < 0.001 for 200 mg and *P* < 0.01 for 150 mg sarilumab dose vs. placebo. Hb, hemoglobin; mTSS, Modified Total Sharp Score; MTX, methotrexate; q2w, every 2 weeks; SD, standard deviation

The mean (SD) change from baseline to weeks 24 and 52 in mTSS in the sarilumab 200 mg, sarilumab 150 mg, and placebo arms is shown in Fig. 4 (stratified by Hb level).

By week 52, the inhibition of progression of structural damage was 84% (sarilumab 200 mg) and 68% (sarilumab 150 mg) vs. placebo in patients with low Hb and 97% (sarilumab 200 mg) and 68% (sarilumab 150 mg) vs. placebo in patients with normal Hb. Overall, the patients who received placebo + MTX had significantly more radiographic progression than the patients in the sarilumab 150 mg and 200 mg + MTX treatment groups, as assessed by mTSS through week 52.

#### Joint space narrowing

The mean (SD) change from baseline to weeks 24 and 52 in JSN in the sarilumab 200 mg, sarilumab 150 mg, and placebo arms is shown in Suppl. Fig. 8 (stratified by Hb level).

By week 52, the inhibition of progression of structural damage was 67.1% (sarilumab 200 mg) and 48.0% (sarilumab 150 mg) vs. placebo in patients with low Hb and 95.1% (sarilumab 200 mg) and 75.4% (sarilumab 150 mg) vs. placebo in patients with normal Hb.

#### Erosion score

The mean (SD) change from baseline to weeks 24 and 52 in erosion score in the sarilumab 200 mg, sarilumab 150 mg, and placebo arms is shown in Suppl. Fig. 9 (stratified by Hb level).

By week 52, the inhibition of progression of structural damage was 95.5% (sarilumab 200 mg) and 81.7% (sarilumab 150 mg) vs. placebo in patients with low Hb and 98.1% (sarilumab 200 mg) and 58.9% (sarilumab 150 mg) vs. placebo in patients with normal Hb.

#### Safety

The safety profile of sarilumab has been previously reported and was not part of this post hoc analysis. In a long-term safety analysis, the most frequent AEs associated with sarilumab were neutropenia, increased alanine aminotransferase, injection site erythema, upper respiratory infections, urinary tract infections, nasopharyngitis, and bronchitis [21, 23, 24].

## Discussion

In these post hoc analyses from baseline to week 24, higher Hb levels were consistently associated with better disease outcomes (CDAI, DAS-28 CRP, pain-VAS, PtGA, and TJC28) in both study groups (pooled TARGET+MOBILITY, MONARCH). These results are in line with other published studies. Hashimoto et al. reported significant increases in Hb levels in anemic RA patients and better clinical outcomes (CDAI and CRP) after TCZ therapy compared with non-biologic therapies even after adjustment for baseline characteristics and baseline disease activity [17].

Strand et al. [26] reported that in sarilumab-treated patients, there was an improvement in PROs such as PtGA, pain-VAS, Stanford Health Assessment Questionnaire-Disability Index (HAQ-DI) score, and FACIT-F after 2 weeks of therapy. This improvement was numerically greater in the sarilumab 200 mg group vs. the sarilumab 150 mg q2w group and was sustained through week 52. Another exploratory analysis reported that at week 24, both sarilumab 150 mg and 200 mg q2w were superior to placebo in all clinical efficacy scores (American College of Rheumatology 20 (ACR20) response, DAS28-CRP remission rate, HAQ-DI score, and CDAI score) [27].

In these analyses, the association between improvement in Hb levels and clinical outcomes was more pronounced among patients treated with sarilumab than those treated with placebo and adalimumab. The findings are similar to those in the published literature [28, 29]. Song et al. [18] reported that in RA patients with anemia, treatment with both tocilizumab and TNF inhibitors (etanercept, infliximab, or adalimumab) resulted in significant improvements in anemia and disease activity, while the effect was more pronounced in patients treated with tocilizumab vs. those treated with TNF inhibitors. An analysis of the Rhumadata® clinical database and registry [30] confirmed that the improvement in anemia observed with IL-6 inhibitors was “numerically and statistically superior” to TNF inhibitors.

IL-6 induces hepcidin which plays a critical role in the development of anemia by causing alterations in iron homeostasis [31, 32]. A recent study addressed the laboratory and hematological parameters associated with RA and more specifically with anemia of chronic disease. The report confirms that patients with RA have a significant elevation of serum IL-6 levels including the patient group with anemia [33]. Our results suggest that the blockade of IL-6 receptor by sarilumab improves the hemoglobin level, possibly via decreasing hepcidin levels, and may contribute to the greater improvement in anemia observed with IL-6 inhibitors [34]. In the in vitro experiments conducted by Song et al., a decrease in serum IL-6 and hepcidin levels was observed in patients treated with TNF inhibitors suggesting that the TNF inhibitors may cause an indirect reduction in hepcidin levels by reducing IL-6 [18].

Although it can be postulated that IL-6 inhibition by sarilumab was effective in increasing Hb levels, independent of the baseline Hb levels, other possibilities cannot be ruled out; for example, IL-6Ri may counter at the target tissue level the inhibitory effects of elevated IL-6 level on erythropoiesis or the erythropoietin response as previously reported [34]. The findings did not address the molecular responses induced by sarilumab on erythropoiesis and therefore cannot fully address the causality between therapy and modification of Hb levels.

In these analyses of phase III randomized controlled trials, dissociation between Hb and clinical efficacy measures was observed by week 24 in sarilumab-treated patients but not in those treated with placebo or adalimumab. These results imply that the positive effect of treatment with sarilumab does not vary by whether a patient has low Hb at baseline.

Preventing joint damage progression and improvement in health-related quality of life are important therapeutic goals in RA [35]. The results showed that patients with low Hb at baseline were at a greater risk for joint damage progression over 52 weeks in the MOBILITY trial than those with normal Hb. Overall, Hb levels could have the potential to act as a surrogate biomarker for predicting the improvement of individual-tailored treatment and choice of drug therapies in RA patients. In the sarilumab + MTX groups, joint damage progression was mitigated compared with placebo + MTX. A similar magnitude of the effect was observed, irrespective of the Hb stratification. These results are consistent with other studies that have demonstrated improvements in long-term radiographic outcomes with early intensive treatment in patients with RA [36–39]. In an open-label extension study, 5-year follow-up data showed that sarilumab + MTX resulted in reductions in disease activity, inhibition of radiographic progression, and improvements in physical function [40].

This study has some limitations. Due to the lack of data on markers of iron status (serum iron, ferritin, and transferrin), hepcidin, or erythropoietin, we could not assess the precise mechanisms of anemia in the included study population. In this post hoc analysis, compensation for repeated analyses was not included in the methodology; as a result, nominal *P* values were generated without formal adjustments for multiple comparisons. Moreover, the limited sample size for patients with low Hb at baseline prevented pursuing stratification by characteristics linked to clinical outcomes or multivariate analysis with adjustment for baseline characteristics as previously reported by Hashimoto et al. [17]. Additional investigations would be required to potentially address these limitations.

## Conclusions

In these post hoc analyses of the three phase III trials of sarilumab, improvements in clinical outcomes for the approved sarilumab 200 mg dose remained independent of changes in Hb level. Clinical, PROs, and laboratory improvements with sarilumab were greater than comparator groups in patients with low Hb levels. Sarilumab treatment was associated with improvement of the clinical outcomes over 24 weeks and mitigation of joint damage progression over 52 weeks, irrespective of baseline Hb levels. By week 24, more sarilumab-treated patients with low Hb levels showed improvement in clinical efficacy measures and PROs than adalimumab-treated patients.

## Supplementary Information

**Additional file 1: Supplementary Table 1.** Concomitant Medications at Baseline. **Supplementary Table 2.** Proportion of Responders* With Improvement in CDAI and Hb at Respective Visit – MONARCH (Safety Population). **Supplementary Table 3.** Proportion of responders* With Improvement in CDAI and Hb at Respective Visit – TARGET + MOBILITY Pooled.

**Additional file 2: Supplementary Figure 1.** Relationship Between Hb Levels and Clinical Efficacy Outcomes: TJC28 and SJC28 Over Time. **Supplementary Figure 2.** Relationship Between Hb Levels and Clinical Efficacy. **Supplementary Figure 3.** Scatterplot of CDAI Against Hb (g/L) by Visit and Baseline Hb Status– MONARCH. **Supplementary Figure 4.** Scatterplots of CDAI Against Hb (g/L) by Visit and Baseline Hb Status – TARGET + MOBILITY Pooled. **Supplementary Figure 5.** Mean Change in Treatment Outcomes: CDAI, DAS28-CRP, Pain-VAS, PtGA, MDGA – TARGET + MOBILITY Pooled. **Supplementary Figure 6.** Mean Change in Treatment Outcomes: TJC28, SJC28, FACIT-Fatigue, and Morning Stiffness – TARGET+MOBILITY Pooled Mean Change in Treatment Outcomes: CDAI, DAS28-CRP, Pain-VAS, PtGA, MDGA – TARGET + MOBILITY Pooled. **Supplementary Figure 7.** Mean Change in Treatment Outcomes: TJC28, SJC28, FACIT-Fatigue, and Morning Stiffness – MONARCH. **Supplementary Figure 8.** Mean change in JSN from Baseline at Week 24 and Week 52 (95% CI) – MOBILITY. **Supplementary Figure 9.** Mean change in Erosion score from Baseline at Week 24 and Week 52 (95% CI) – MOBILITY.

## Data Availability

Qualified researchers may request access to patient-level data and related study documents including the clinical study report, study protocol with any amendments, blank case report form, statistical analysis plan, and data set specifications. Patient-level data will be anonymized, and study documents will be redacted to protect the privacy of our trial participants. Further details on Sanofi’s data sharing criteria, eligible studies, and process for requesting access can be found at https://www.vivli.org/.

## Abbreviations

ACR: American College of Rheumatology
ANCOVA: Analysis of covariance
ART: Arthritis Research and Therapy
BMI: Body mass index
CCP: Cyclic citrullinated peptide
CDAI: Clinical Disease Activity Index
CI: Confidence interval
CRP: C-reactive protein
csDMARD: Conventional synthetic disease-modifying antirheumatic drug
DAS: Disease Activity Scores
DAS28-CRP: Disease Activity Score-28 for Rheumatoid Arthritis with CRP
DMARD: Disease-modifying antirheumatic drug
FACIT-F: Functional Assessment of Chronic Illness Therapy-Fatigue
GABA: Gamma-aminobutyric acid
HAQ-DI: Stanford Health Assessment Questionnaire-Disability Index
Hb: Hemoglobin
IL: Interleukin
JSN: Joint Space Narrowing Score
MDGA: Physician Global Assessment
mTSS: Modified Total Sharp Score
MTX: Methotrexate
*N*: Total number of patients in each study group with low or normal Hb subgroup
*n*: Number of patients in each subgroup
NSAID: Nonsteroidal anti-inflammatory drug
*P* value: Probability
Pain-VAS: Pain as assessed by the visual analog scale
PRO: Patient-reported outcome
PtGA: Patient Global Assessment
q2w: Every 2 weeks
RA: Rheumatoid arthritis
SD: Standard deviation
SJC28: Swollen 28-Joint Count
SSRI: Selective serotonin reuptake inhibitor
TCZ: Tocilizumab
TJC28: Tender 28-Joint Count
TNF: Tumor necrosis factor
TNFi: Tumor necrosis factor inhibitor
VAS: Visual analog scale
WHO: World Health Organization
