# Supplementary material for: Association between low hemoglobin, clinical measures, and patient-reported outcomes in patients with rheumatoid arthritis: results from post hoc analyses of three phase III trials of sarilumab
Source: Arthritis Res Ther. 2022 Aug 25;24:207. doi: 10.1186/s13075-022-02891-x (PMC9404615; doi:10.1186/s13075-022-02891-x)
Supplement: Supplementary file 1 — Additional file 1: Supplementary Table 1. Concomitant Medications at Baseline. Supplementary Table 2. Proportion of Responders* With Improvement in CDAI and Hb at Respective Visit – MONARCH (Safety Population). Supplementary Table 3. Proportion of responders* With Improvement in CDAI and Hb at Respective Visit – TARGET + MOBILITY Pooled. [file 13075_2022_2891_MOESM1_ESM.docx]

# **Supplementary Tables**

**Supplementary Table 1. Concomitant Medications at Baseline**

|  | TARGET | | MOBILITY | | MONARCH | |
| --- | --- | --- | --- | --- | --- | --- |
| **Concomitant medications** | **Low Hb (N = 186)** | **Normal Hb (N = 360)** | **Low Hb (N = 414)** | **Normal Hb (N = 783)** | **Low Hb (N = 92)** | **Normal Hb (N = 277)** |
| Opioid use, n (%) | 42 (22.6) | 57 (15.8) | 48 (11.6) | 87 (11.1) | 13 (14.1) | 25 (9.0) |
| Corticosteroid use, n (%) | 124 (66.7) | 217 (60.3) | 250 (60.4) | 505 (64.5) | 52 (56.5) | 150 (54.2) |
| NSAID use, n (%) | 132 (71.0) | 250 (69.4) | 301 (72.7) | 503 (64.2) | 60 (65.2) | 181 (65.3) |
| SSRI use, n (%) | 5 (2.7) | 18 (5.0) | 12 (2.9) | 34 (4.3) | 1 (1.1) | 4 (1.4) |
| GABA use^*^, n (%) | 5 (2.7) | 8 (2.2) | 5 (1.2) | 7 (0.9) | 1 (1.1) | 2 (0.7) |
| Neutrophil count, 10^9/L, mean (SD) | 6.4 (2.5) | 6.0 (2.5) | 6.2 (2.5) | 6.0 (2.4) | 5.7 (2.5) | 5.6 (2.3) |

N = Number of patients in each study group with low or normal Hb subgroup.

^*^Includes gabapentin (Neurontin) and pregabalin (Lyrica) in TARGET; gabapentin (Neurontin), pregabalin (Lyrica) and Newgaba in MOBILITY; Gabapentin, Lyrica, Gabagamma, Gabanox, Pragiola, and pregabalin in MONARCH.

GABA, gamma-aminobutyric acid; Hb, hemoglobin; NSAIDs, nonsteroidal anti-inflammatory drugs; SD, standard deviation; SSRIs, selective serotonin reuptake inhibitors.

**Supplementary Table 2. Proportion of Responders* With Improvement in CDAI and Hb at Respective Visit – MONARCH (Safety Population)**

|  | | **Sarilumab 200mg Q2W (N = 184)** | **Adalimumab 40mg Q2W (N = 184)** |
| --- | --- | --- | --- |
|  | Week 4 | | |
| Number | | 179 | 172 |
| Responders | | 36 (20.1%) | 41 (23.8%) |
| Non-responders | | 143 (79.9%) | 131 (76.6%) |
|  | Week 8 | | |
| Number | | 173 | 169 |
| Responders | | 84 (48.6%) | 68 (40.2%) |
| Non-responders | | 89 (51.5%) | 101 (59.8%) |
|  | Week 12 | | |
| Number | | 165 | 170 |
| Responders | | 94 (57.0%) | 82 (48.2%) |
| Non-responders | | 71 (43.0%) | 88 (51.8%) |
|  | Week 20 | | |
| Number | | 162 | 158 |
| Responders | | 111 (68.5%) | 93 (58.9%) |
| Non-responders | | 51 (31.5%) | 65 (41.1%) |
|  | Week 24 | | |
| Number | | 156 | 148 |
| Responders | | 111 (71.2%) | 90 (60.8%) |
| Non-responders | | 45 (28.9%) | 58 (39.2%) |
| CDAI:  TJC28 + SJC28 + Patient global VAS + Physician global VAS ^*^Responder: CDAI improvement from baseline of ≥ 50% and Hb ≥120 g/L for women or Hb ≥130 g/L for men N: Number of patients within each treatment arm. Number: Number of patients with non-missing CDAI and Hb values within each treatment arm at respective visit.  CDAI, Clinical Disease Activity Index; Hb, hemoglobin; q2w, every 2 weeks; VAS, visual analog scale. | | | |

**Supplementary Table 3. Proportion of responders* With Improvement in CDAI and Hb at Respective Visit – TARGET + MOBILITY Pooled**

|  | **Placebo (N = 578)** | **Sarilumab 150mg Q2W (N = 582)** | **Sarilumab 200mg Q2W (N = 580)** |
| --- | --- | --- | --- |
| Week 4 | | | |
| Number | 554 | 545 | 556 |
| Responders | 79 (14.3%) | 143 (26.2%) | 144 (25.9%) |
| Non-responders | 475 (85.7%) | 402 (73.8%) | 412 (74.1%) |
| Week 8 | | | |
| Number | 535 | 537 | 539 |
| Responders | 105 (19.6%) | 212 (39.5%) | 246 (45.6%) |
| Non-responders | 430 (80.4%) | 325 (60.5%) | 293 (54.4%) |
| Week 12 | | | |
| Number | 531 | 520 | 524 |
| Responders | 128 (24.1%) | 232 (44.6%) | 278 (53.1%) |
| Non-responders | 403 (75.9%) | 288 (55.4%) | 246 (47.0%) |
| Week 20 | | | |
| Number | 380 | 461 | 451 |
| Responders | 140 (36.8%) | 281 (61.0%) | 297 (65.9%) |
| Non-responders | 240 (63.2%) | 180 (39.1%) | 154 (34.2%) |
| Week 24 | | | |
| Number | 338 | 429 | 437 |
| Responders | 145 (42.9%) | 277 (64.6%) | 313 (71.6%) |
| Non-responders | 193 (57.1%) | 152 (35.4%) | 124 (28.4%) |
| CDAI: TJC28 + SJC28 + Patient global VAS + Physician global VAS ^*^Responder: CDAI improvement from baseline of ≥ 50% and Hb ≥ 120 g/L for women or Hb ≥ 130 g/L for men N: Number of patients within each treatment arm. Number: Number of patients with nonmissing CDAI and Hb values within each treatment arm at respective visit. | | | |

CDAI, Clinical Disease Activity Index; Hb, hemoglobin; q2w, every 2 weeks; VAS, visual analog scale.
