# Supplementary material for: Association between low hemoglobin, clinical measures, and patient-reported outcomes in patients with rheumatoid arthritis: results from post hoc analyses of three phase III trials of sarilumab
Source: Arthritis Res Ther. 2022 Aug 25;24:207. doi: 10.1186/s13075-022-02891-x (PMC9404615; doi:10.1186/s13075-022-02891-x)
Supplement: Supplementary file 2 — Additional file 2: Supplementary Figure 1. Relationship Between Hb Levels and Clinical Efficacy Outcomes: TJC28 and SJC28 Over Time. Supplementary Figure 2. Relationship Between Hb Levels and Clinical Efficacy. Supplementary Figure 3. Scatterplot of CDAI Against Hb (g/L) by Visit and Baseline Hb Status– MONARCH. Supplementary Figure 4. Scatterplots of CDAI Against Hb (g/L) by Visit and Baseline Hb Status – TARGET + MOBILITY Pooled. Supplementary Figure 5. Mean Change in Treatment Outcomes: CDAI, DAS28-CRP, Pain-VAS, PtGA, MDGA – TARGET + MOBILITY Pooled. Supplementary Figure 6. Mean Change in Treatment Outcomes: TJC28, SJC28, FACIT-Fatigue, and Morning Stiffness – TARGET+MOBILITY Pooled Mean Change in Treatment Outcomes: CDAI, DAS28-CRP, Pain-VAS, PtGA, MDGA – TARGET + MOBILITY Pooled. Supplementary Figure 7. Mean Change in Treatment Outcomes: TJC28, SJC28, FACIT-Fatigue, and Morning Stiffness – MONARCH. Supplementary Figure 8. Mean change in JSN from Baseline at Week 24 and Week 52 (95% CI) – MOBILITY. Supplementary Figure 9. Mean change in Erosion score from Baseline at Week 24 and Week 52 (95% CI) – MOBILITY. [file 13075_2022_2891_MOESM2_ESM.docx]

# **Supplementary Figures**


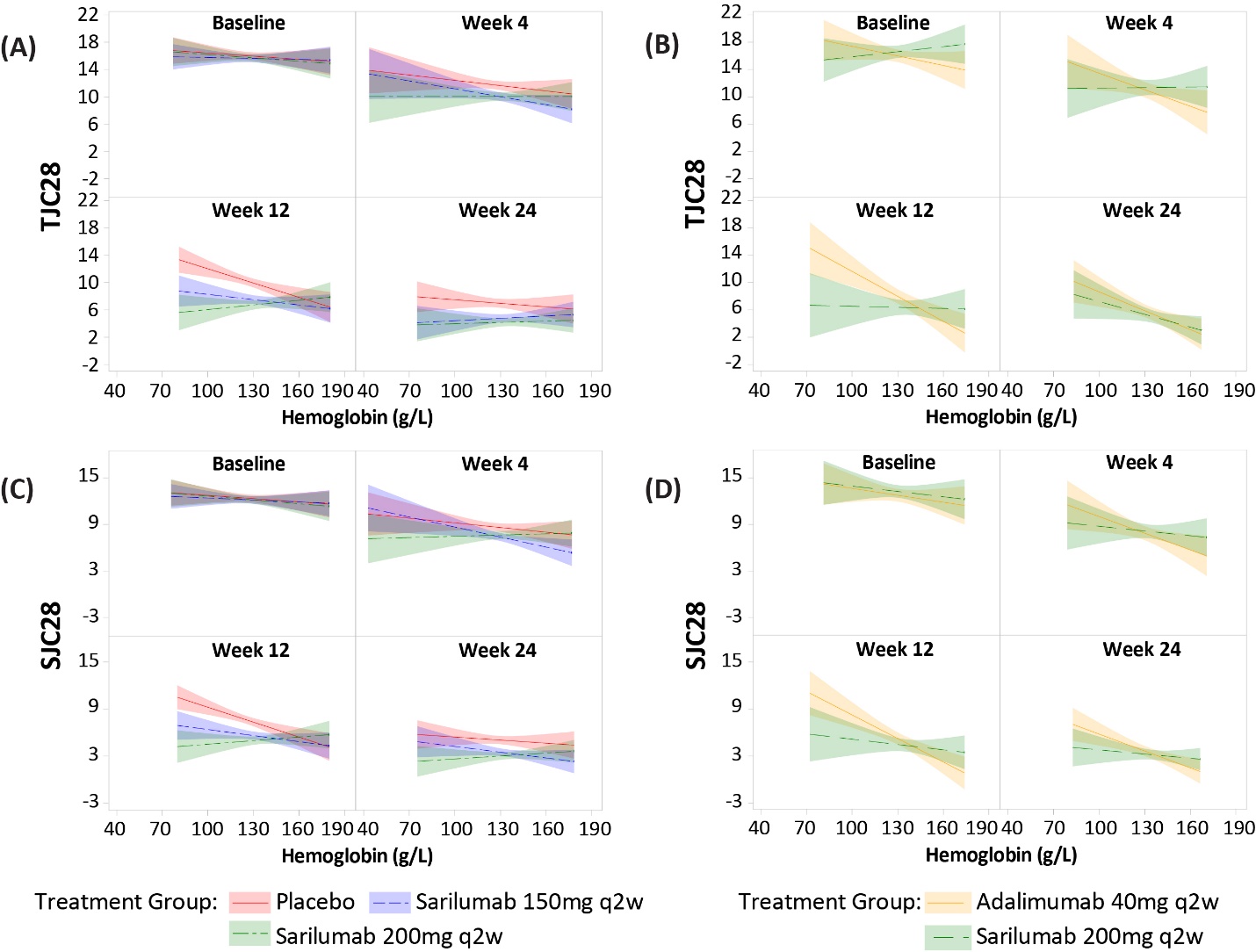
**Supplementary Figure 1: Relationship Between Hb Levels and Clinical Efficacy Outcomes: TJC28 and SJC28 Over Time**

**MONARCH**

**TARGET + MOBILITY Pooled**

Hb, hemoglobin; q2w, every 2 weeks; SD, standard deviation; SJC28, Swollen 28-Joint Count; TJC28, Tender 28-Joint Count.


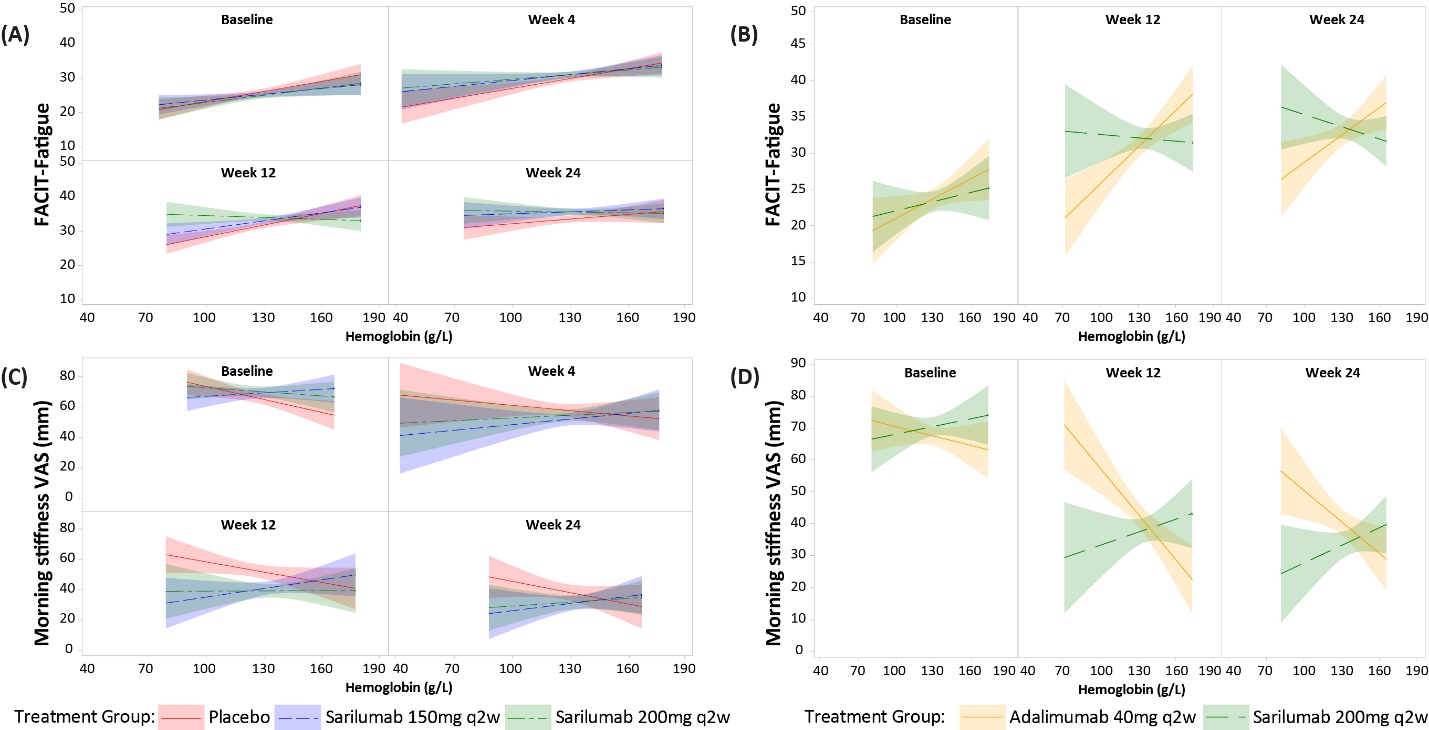
**Supplementary Figure 2: Relationship Between Hb Levels and Clinical Efficacy Outcomes: FACIT-F and Morning Stiffness (VAS) Over Time**

**MONARCH**

**TARGET + MOBILITY Pooled**

FACIT-F, Functional Assessment of Chronic Illness Therapy – Fatigue; Hb, hemoglobin; q2w, every 2 weeks; Morning Stiffness-VAS, Morning Stiffness assessed by visual analogue scale; SD, standard deviation

**Supplementary Figure 3: Scatterplot of CDAI Against Hb (g/L) by Visit and Baseline Hb Status– MONARCH**


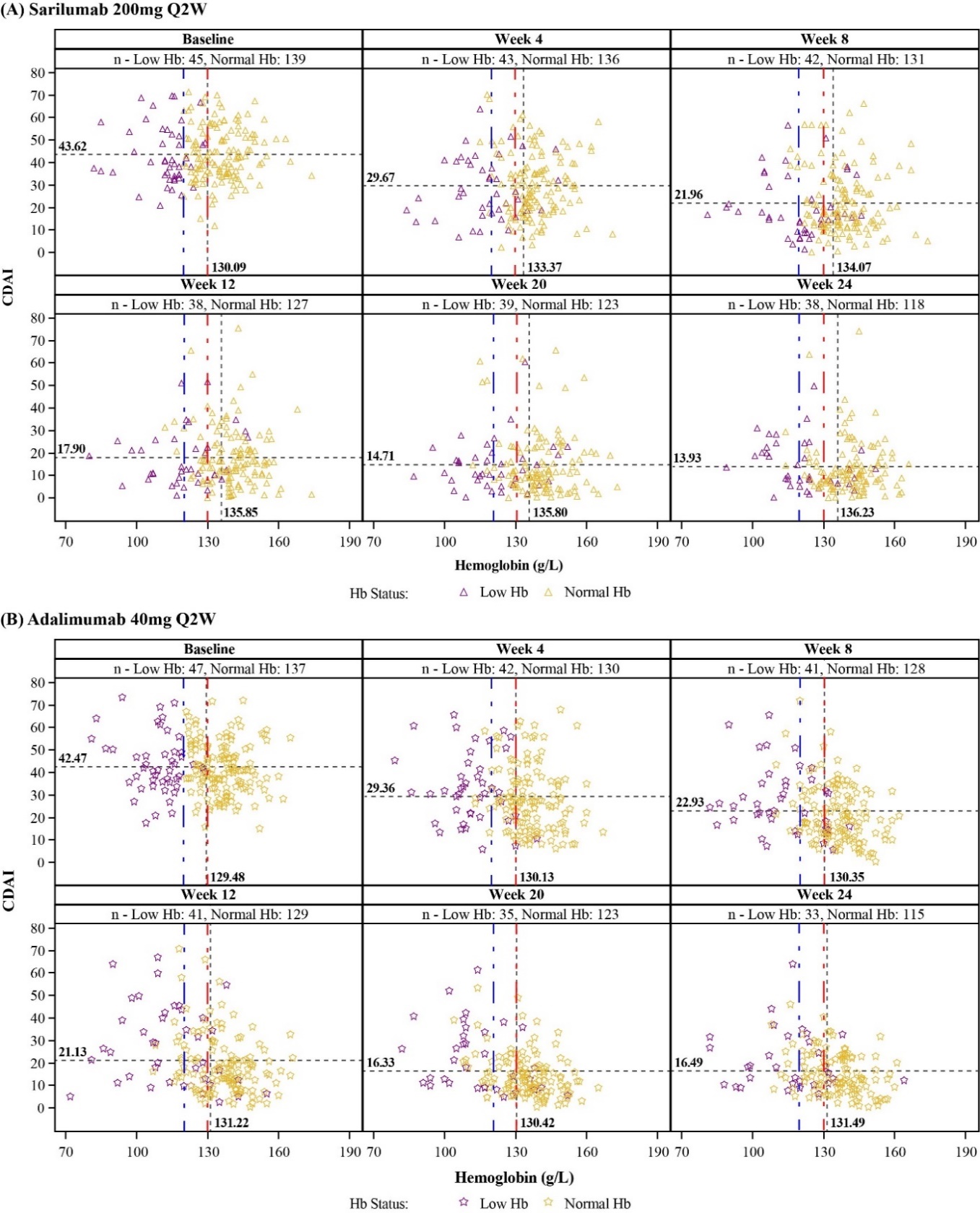
CDAI: TJC28 + SJC28 + Patient global VAS + Physician global VAS. n: Number of patients within each treatment arm and Hb subgroup at respective week. Patients with non-missing CDAI and Hb (g/L) values are considered. Blue and red reference vertical lines correspond to normal Hb (WHO cut-off) for females and males, respectively. Baseline Hb <120 g/L for women or <130 g/L for men is considered as low Hb; whereas baseline Hb ≥120 g/L for women or ≥130 g/L for men is considered as Normal Hb as per WHO guidelines. Patients with missing Hb at baseline are considered under Normal Hb status. Horizontal and vertical reference lines correspond to mean value of CDAI and Hb at respective visit. Blue and red reference lines correspond to Normal Hb (WHO cut off) for females and males respectively.

CDAI, Clinical Disease Activity Index; Hb, hemoglobin; SJC28, Swollen 28-Joint Count; TJC28, Tender 28- Joint Count; VAS, visual analogue scale; WHO, World Health Organization.

**Supplementary Figure 4: Scatterplots of CDAI Against Hb (g/L) by Visit and Baseline Hb Status – TARGET + MOBILITY Pooled**


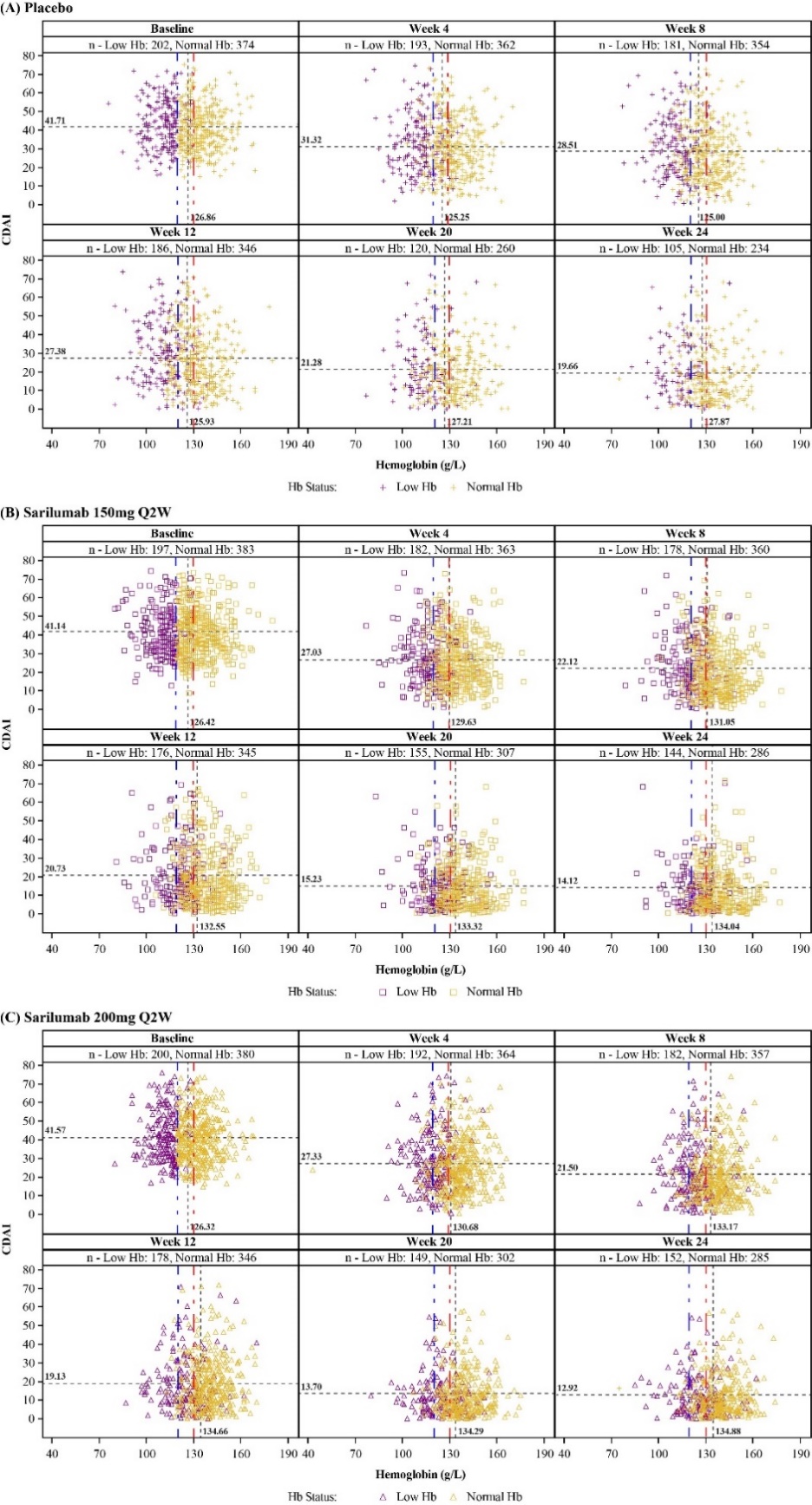


CDAI: TJC28 + SJC28 + Patient global VAS + Physician global VAS. n: Number of patients within each treatment arm and Hb subgroup at respective week. Patients with non-missing CDAI and Hb (g/L) values are considered. Baseline Hb <120 g/L for women or <130 g/L for men is considered as low Hb; whereas baseline Hb ≥120 g/L for women or ≥130 g/L for men is considered as Normal Hb as per WHO guidelines. Patients with missing Hb at baseline are considered under Normal Hb status. Horizontal and vertical reference lines correspond to mean value of CDAI and Hb at respective visit. Blue and red reference lines correspond to Normal Hb (WHO cut off) for females and males respectively.

CDAI, Clinical Disease Activity Index; Hb, hemoglobin; SJC28, Swollen 28-Joint Count; q2w, every 2 weeks; TJC28, Tender 28- Joint Count; VAS, visual analogue scale; WHO, World Health Organization.

**
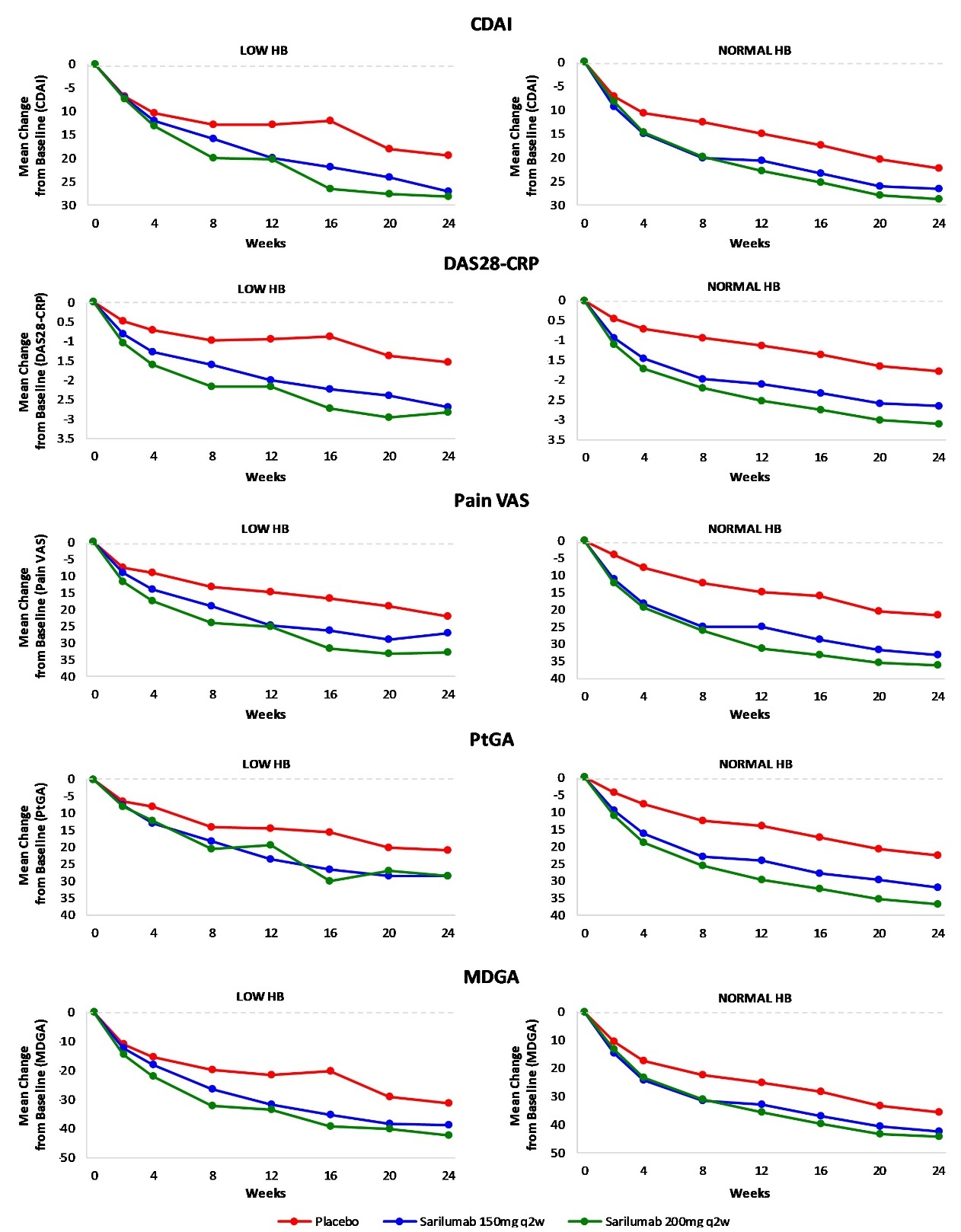
Supplementary Figure 5: Mean Change in Treatment Outcomes: CDAI, DAS28-CRP, Pain-VAS, PtGA, MDGA – TARGET + MOBILITY Pooled**

CDAI, Clinical Disease Activity Index; CRP, C-reactive protein; DAS28-CRP, Disease Activity Score-28 for Rheumatoid Arthritis with CRP; Hb, hemoglobin; PtGA, Patient Global Assessment; MDGA, Physician Global Assessment; q2w, every 2 weeks; VAS, visual analogue scale.

**Supplementary Figure 6: Mean Change in Treatment Outcomes: TJC28, SJC28, FACIT-Fatigue, and Morning Stiffness – TARGET+MOBILITY Pooled**


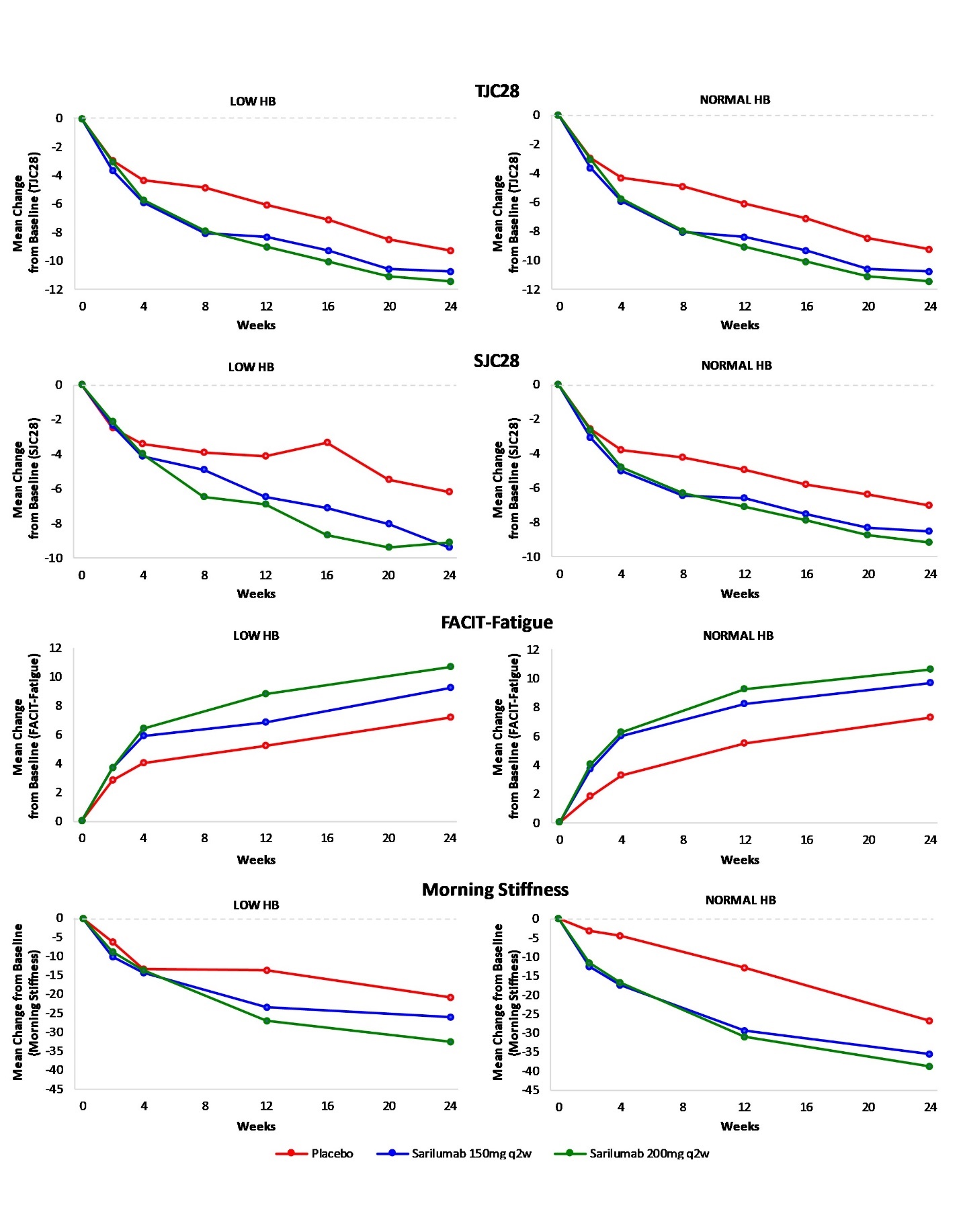


FACIT-F, Functional Assessment of Chronic Illness Therapy-Fatigue; Hb, hemoglobin; q2w, every 2 weeks; SJC28, Swollen 28-Joint Count; TJC28, Tender 28- Joint Count; VAS, visual analogue scale.

**Supplementary Figure 7: Mean Change in Treatment Outcomes: TJC28, SJC28, FACIT-Fatigue, and Morning Stiffness – MONARCH**


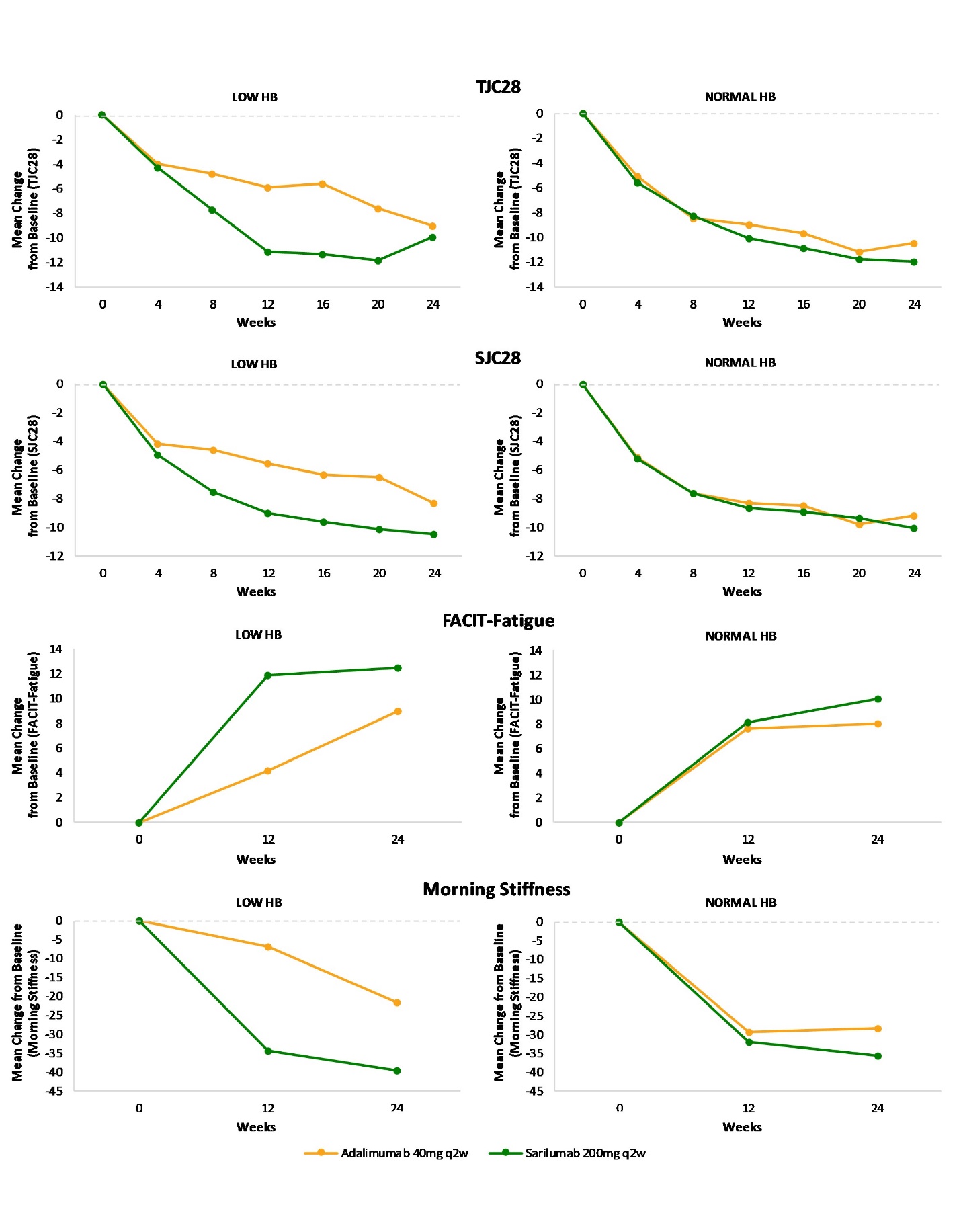


FACIT-F, Functional Assessment of Chronic Illness Therapy-Fatigue; Hb, hemoglobin; q2w, every 2 weeks; SJC28, Swollen 28-Joint Count; TJC28, tender 28- joint count; VAS, visual analogue scale.

**Supplementary Figure 8: Mean change in JSN from Baseline at Week 24 and Week 52 (95% CI) – MOBILITY**

**
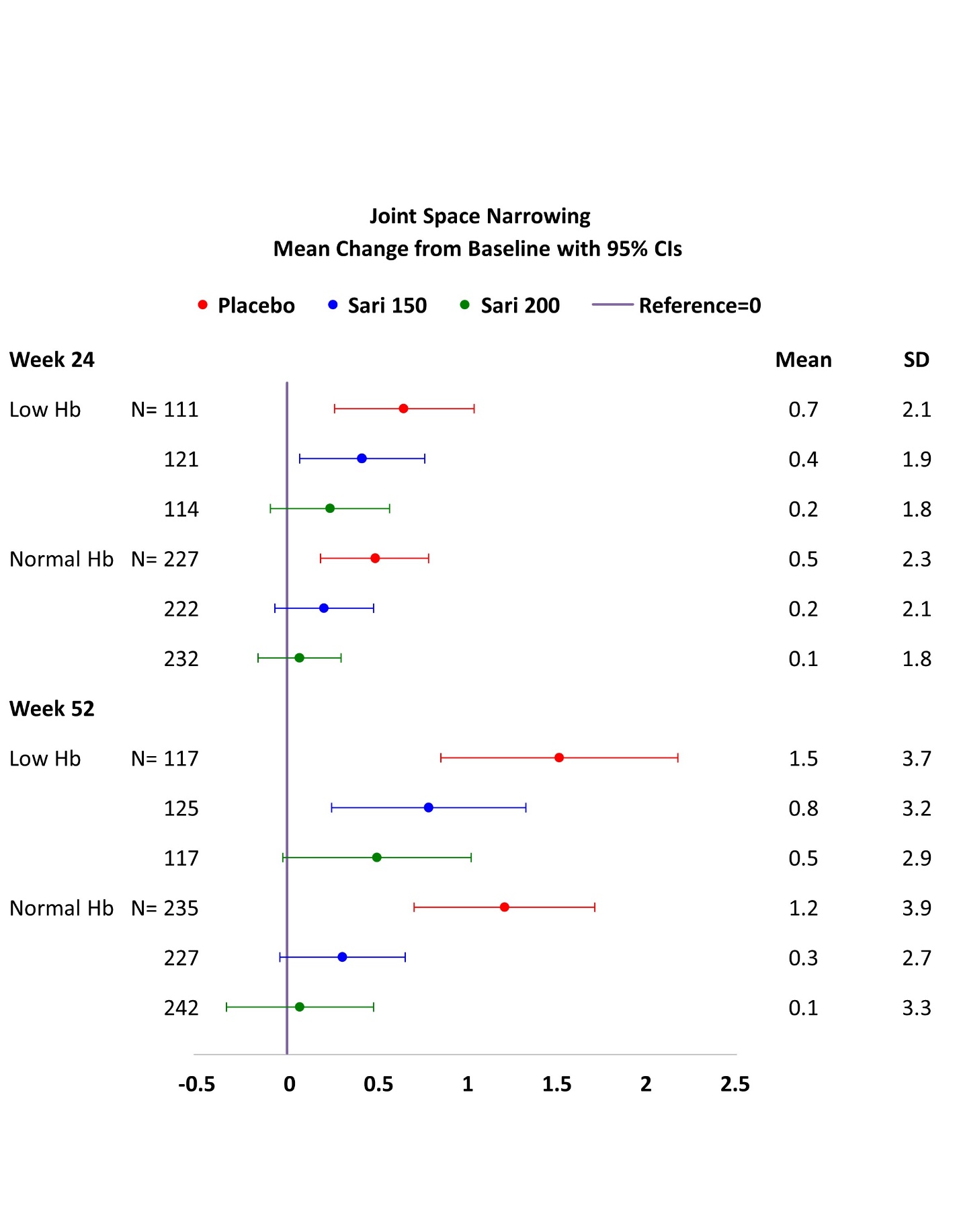
**All patients received weekly MTX and sarilumab or placebo were administered q2w. At week 52: Low Hb: Rank ANCOVA *P*<0.01 for 200 mg and *P*<0.05 for 150 mg sarilumab dose vs placebo; Normal Hb: Rank ANCOVA *P*<0.001 for 200 mg and *P*<0.01 for 150 mg sarilumab dose vs placebo.

Hb, hemoglobin; JSN, joint space narrowing score; MTX, methotrexate; q2w, every 2 weeks; SD, standard deviation.

**Supplementary Figure 9. Mean change in Erosion score from Baseline at Week 24 and Week 52 (95% CI) – MOBILITY**

**
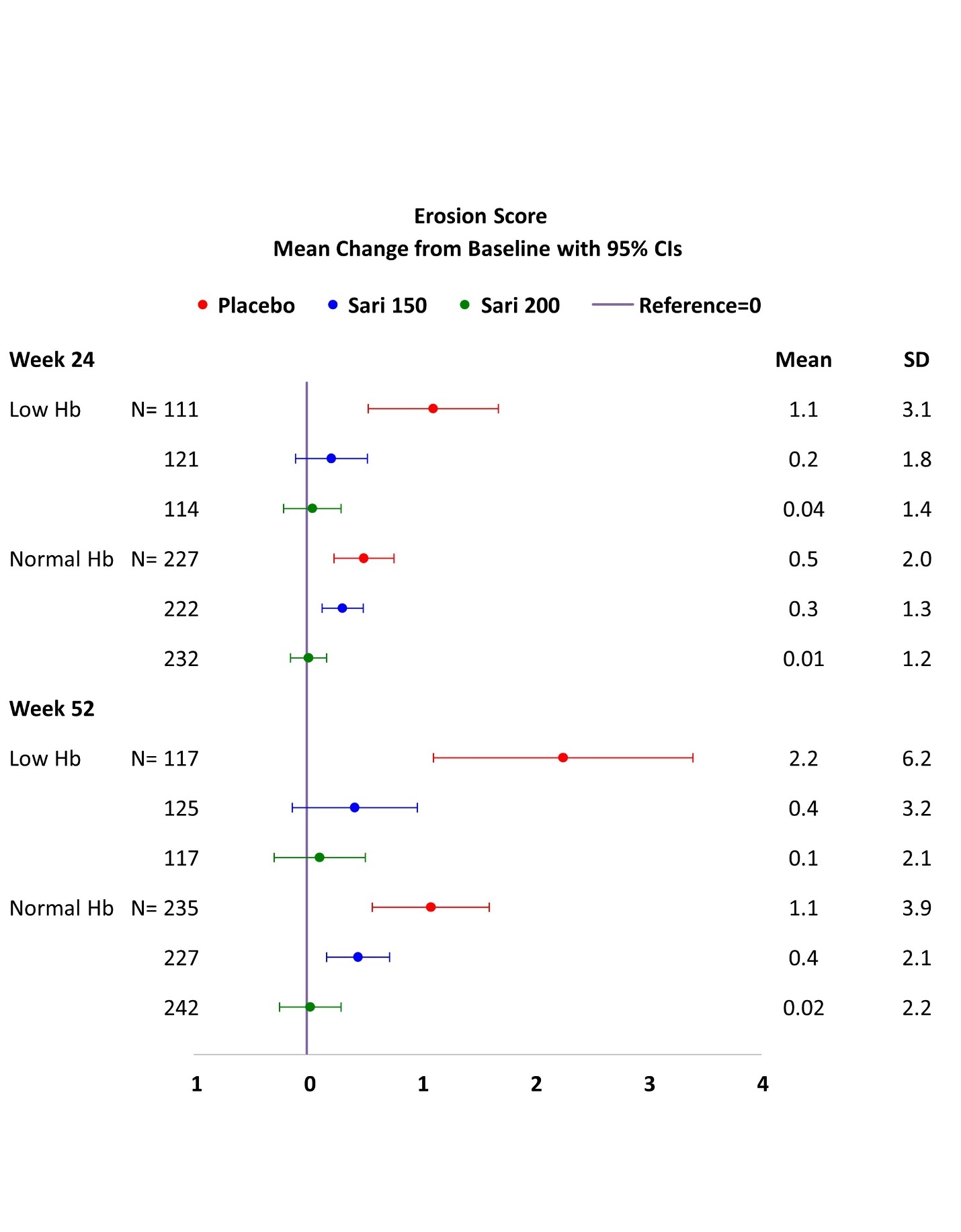
**All patients received weekly MTX and sarilumab or placebo were administered q2w. At week 52: Low Hb: *P*<0.001 by rank ANCOVA for each sarilumab dose vs placebo; Normal Hb: Rank ANCOVA *P*<0.001 for 200 mg and *P*<0.05 for 150 mg sarilumab dose vs placebo.

Hb, hemoglobin; MTX, methotrexate; q2w, every 2 weeks; SD, standard deviation.
